# High school student cannabis use and perceptions towards cannabis in southcentral Colorado – comparing communities that permit recreational dispensaries and communities that do not

**DOI:** 10.1186/s42238-019-0002-0

**Published:** 2019-06-07

**Authors:** Tim Peters, Carol Foust

**Affiliations:** Colorado State University Pueblo, 2200 Bonforte Blvd, Pueblo, CO 81001 USA

**Keywords:** Youth marijuana use, Perceptions, Cannabis, Legalization, Recreational dispensaries, ANOVA

## Abstract

Currently, with the legalization of cannabis and the opening of recreational dispensaries in states across the country, the question of whether or not proximity to recreational cannabis dispensaries affects high school students in terms of their cannabis use, their perceptions of the accessibility of cannabis and their perceptions on the harmfulness and wrongfulness of using cannabis is particularly relevant and timely. In 2014 in Colorado, Amendment 64 went into effect and communities were allowed to legally permit recreational cannabis dispensaries; some communities agreed to permit the opening of recreational dispensaries while other communities did not. Using data from the cross-sectional Healthy Kids Colorado Survey collected from students in randomly selected high schools in both 2013 and 2015, data on student use and perceptions towards cannabis use was analyzed comparing communities that permitted recreational cannabis dispensaries and communities that did not.

The random cross-sectional design used a 2X2 factorial ANOVA for each of the dependent factors: use, access, wrongfulness, and harm. There were a total of three communities that permitted recreational dispensaries, and within those three communities, data was collected from seven high schools. There were four communities that did permit recreational dispensaries, and within those four communities, data was collected from five high schools. The data were aggregated into two groups: ‘yes’ allows dispensaries, and ‘no’ does not allow dispensaries. These two groups were used as comparisons in the factorial ANOVA along with the two collection event years of 2013 and 2015.

The analysis indicates differences between students in communities that have never permitted recreational cannabis dispensaries and students in communities that opened recreational dispensaries in 2014. Students in communities that permitted recreational dispensaries used more cannabis, thought cannabis was less harmful, less wrong, and was more difficult to access than high school students in communities that did not permit recreational cannabis dispensaries, however these differences existed before and after recreational dispensaries were introduced in 2014.

Looking at each type of community to see if there was a change between 2013 and 2015, there were no statistically significant differences between students in 2013 and 2015 in each type of community with one exception; students in communities that did not permit recreational cannabis dispensaries felt even more strongly in 2015 that cannabis use is wrong compared to 2013. Based on the 2013 and 2015 Healthy Kids Colorado Survey data, permitting or not permitting recreational cannabis dispensaries in a community does not appear to change student cannabis use or perceptions towards cannabis.

## Background

In November 2012, Colorado voters passed Amendment 64, which legalized recreational cannabis for adults 21 years and older. The ballot measure allowed for the licensing of retail stores, or dispensaries, whose purpose was the legal distribution of recreational cannabis. The Amendment also gave local governments the power to regulate or prohibit such facilities in their local jurisdiction.

Five years later, debates surrounding the effects of legalized recreational cannabis grow as more and more local governments in Colorado face the choice of whether or not recreational cannabis dispensaries should be allowed in their jurisdiction. In 2016, fifteen municipalities across Colorado held ballot measures related to cannabis regulation (Mooney 2016). Eight communities banned recreational cannabis sales and seven communities permitted recreational sales.

Similar debates are not just occurring in Colorado but are occurring across the United States. In 2016, California, Maine, Massachusetts, and Nevada joined Colorado, Washington, Oregon and Alaska in passing legislation to permit the sale of recreational cannabis. Each state has given local governments the authority to regulate retail cannabis stores. From Roseville, California (Westrope 2017) to Braintree, Massachusetts’s (Hinckley 2016) local governments are debating whether or not to permit recreational cannabis dispensaries in their communities. Debates about the pros and cons of permitting cannabis dispensaries in a community continue to grow as 14 more states currently have policy makers drafting legislation proposing cannabis legalization (Wilder 2017).

An argument made by the proponents of allowing retail cannabis dispensaries is the economic impact of cannabis sales. The Marijuana Policy Group, a collaborative effort between researchers at the University of Colorado Business Research Division and the firm BBC Research and Consulting, found that in Colorado, in just the year 2015, cannabis sales totaled $996 million, generating $2.39 billion in economic impact, and creating over 18,000 new Full-Time-Equivalent (FTE) positions. The report claims that demand is expected to grow by 11.3% per year through 2020 (Light et al. 2016). At the local level, Pueblo County in southern Colorado generated $763,680 in tax revenues from recreational cannabis sales in 2016 and Denver County reported over $8.2 million in recreational cannabis tax revenue (State of Colorado 2017).

Advocates for permitting recreational sales also argue the libertarian belief of self-ownership. Individuals, not government, they argue, should determine what people do with their bodies (Wilson 2015). Furthermore, many advocates for legalization state how the impact of regulated legal sales will undercut the black market and criminal activities associated with cannabis in a community (Morris et al. 2014).

Opponents argue against permitting recreational cannabis dispensaries due to the potential negative social impact of these stores. In 2016, Pueblo County ballot Question 200 proposed a repeal of ordinances allowing recreational cannabis sales. Supporters of the Ballot measure said that the recreational cannabis industry had caused an increase in the transient population, higher crime rates, increased emergency room visits, and an unwanted stigma (Wallace 2016). Possibly the biggest concern raised by supporters of the ballot initiative is the effects that legalizing recreational cannabis for adults has on cannabis use by youth.

Several studies have measured youth cannabis use before and after legalized recreational cannabis in Colorado. Brooks-Russell et al. (Brooks-Russell et al. 2017) in 2017 found that adolescent cannabis use did not increase from 2013 to 2015 despite the opening of recreational cannabis dispensaries across the state in 2014. The authors found a “lack of difference in change by poverty status, minority status, urbanicity, or local policy permitting recreational sales”. In a 2018 study, Brooks et al. (Brooks-Russell et al. 2018) also measured adolescent attitudes towards cannabis, including perceived ease of access, perceived wrongfulness of personal use, and perceived risk of harm from regular cannabis use. Brooks-Russell et al. reported that neither perceived ease of access, nor perceived wrongfulness of personal use changed from 2013 to 2015. However, students’ self-reported perception of the risk of harm from regular cannabis use declined.

Harpin et al. (Harpin et al. 2018) found no relationship between adolescent cannabis use and density of recreational cannabis businesses within 5 miles of schools. The authors mapped 219 recreational cannabis dispensaries and schools. Using the cross-sectional data from the Healthy Kids Colorado Survey (HKCS), they also found that proximity to recreational dispensaries did not significantly contribute to youth’s perception of the ease of access to cannabis.

Studies from outside of Colorado have focused on changes in youth cannabis use when medical cannabis laws were passed as well. It is possible to speculate that results collected after recreational cannabis legalization may mirror results collected after medicinal cannabis was legalized in a state. Johnson, Hodgkin and Harris (Johnson et al. 2017), in a study of 45 states between 1991 and 2011, found that adolescents living in states with medical cannabis laws had higher past 30-day cannabis use compared to those living in states that did not allow medical cannabis; however, they found no evidence of an increase in adolescent past 30-day cannabis use after enactment of medical cannabis laws. Moreover, the study found that enactment of a medical cannabis law appeared to lessen the odds of adolescent cannabis use. Hasin et al. (Hasin et al. 2015) analyzed data from annual, repeated cross-sectional surveys and similarly did not find that medicinal cannabis laws significantly change adolescent cannabis use.

### Our study

This study sought to answer the question “does permitting recreational cannabis dispensaries in a community effect high school students’ cannabis use, their perceptions of the accessibility of cannabis, and their perceptions of the harmfulness and wrongfulness of using cannabis?” A cross-sectional survey of high school students was administered in 2013, before recreational cannabis dispensaries were permitted, and the survey was administered in 2015 at the same high schools but not necessarily the same students, after recreational dispensaries were opened. During those two years some communities had locally permitted recreational cannabis dispensaries and others had not. The 2013 and 2015 data on student cannabis use and perceptions toward cannabis was analyzed to compare high school student use and perceptions in communities in southcentral Colorado that had permitted recreational cannabis dispensaries with high school students in those communities that had not permitted dispensaries.

## Methods

### Sample

This study used cross-sectional data from separate samples of Colorado high school students collected from the Healthy Kids Colorado Survey (HKCS) of 2013 and 2015. HKCS is a cooperative effort between the Colorado Department of Public Health and Environment, the Colorado Department of Education, and the Colorado Department of Human Services. This survey of secondary students in Colorado is given every two years and has included questions on cannabis since 1999. The HKCS is conducted using methods consistent with the Centers for Disease Control and Prevention’s Youth Risk Behavior Survey. Surveys were completed by students from a random sample of selected schools from different regions of the State and randomly selected classrooms within those schools. HKCS is completely voluntary. Districts decide whether or not to participate and then schools within that district decide whether or not the school will participate. Furthermore, parents and children also decide whether the individual student participates. Across the state, 15,970 students from 127 high schools participated in the 2015 state sample. In 2013, statewide 25,197 students and 106 schools participated. Overall response rate is the product of the school participation rate and the student response rate. There was an overall response rate of 47% for high schools in 2015 and 58% in 2013.

Our study focused solely on schools in southcentral Colorado. The survey intended to include schools from both southcentral and southeastern Colorado, but no schools participated in both years from the southeastern corner of the state. The data used was collected from high schools in Pueblo, Teller, Park, Fremont, Alamosa, Chafee and Conejos Counties. Schools were selected based on their geographic location (southcentral Colorado) and whether the school participated in the survey in both 2013 and 2015. Our study included 7 different communities. Three communities permitted and opened recreational cannabis dispensaries in 2014; four communities did not permit recreational cannabis dispensaries. Our HKCS data was collected from 12 high schools (*n* = 3649 in 2013 and *n* = 2696 in 2015). The high schools were in communities that permitted recreational cannabis dispensaries in 2014 (*n* = 2053 in 2013 and *n* = 1328 in 2015) and communities that have never permitted recreational cannabis dispensaries (*n* = 1596 in 2013 and *n* = 1368 in 2015). All schools in this study participated in the cross-sectional HKCS study in both 2013 and 2015 but the individual students did not necessarily participate in both years.

Students completed self-administered machine-readable questionnaires during a regular class period. Participation was confidential. There were two different modules of the test, A and B that were administered in both 2013 and 2015. Module A included questions on cannabis use and perceptions towards ease of access, harmfulness and wrongfulness, Module B asked about student use but did not ask questions related to perceptions towards cannabis. Approximately half of the students completed Module A and half Module B, meaning all students were asked about cannabis use and only about half were asked about perceptions towards ease of access, harmfulness and wrongfulness.

### Study measures

To measure student use, students were asked, “During the past 30 days, how many times did you use marijuana?” Students were given the option to select 6 responses: 0 times, 1 or 2 times, 3 to 9 times, 10–19 times, 20–39 times, or 40 or more times. Students’ perception towards cannabis included questions on the ease of access to cannabis, the perceived harm of cannabis, and the perceived wrongfulness of cannabis use. To measure ease of access, students were asked “If you wanted to get some marijuana, how easy would it be for you to get some?” Students could answer, very hard, sort of hard, sort of easy, and very easy. To measure perceived harmfulness, students were asked, “How much do you think people risk harming themselves (physically or in other ways), if they use marijuana regularly?” Students could respond, no risk, slight risk, moderate risk and great risk. A fourth question asked, “How wrong do you think it is for someone your age to use marijuana?” Students could answer, very wrong, wrong, a little bit wrong, and not wrong at all.

### Analysis

The random cross-sectional design used a 2X2 factorial ANOVA for each of the dependent factors: use, access, wrongfulness, and harm. A cluster random sampling method was used to select schools in each region of the state. Within each school, random classrooms were selected to collect the survey data. The data from schools within communities that allow cannabis dispensaries were aggregated as were the data from schools within communities that did not allow dispensaries. There were a total of three communities that included seven high schools that allowed dispensaries and four communities that included five high schools that did not allow dispensaries. The data were aggregated into two groups: ‘yes’ allows dispensaries, and ‘no’ does not allow dispensaries. These two groups were used as comparisons in the factorial ANOVA along with the two collection event years of 2013 and 2015. The groups were defined as 1) high school students attending schools in 2013 in communities that have never allowed recreational cannabis dispensaries, 2) high school students in 2013 attending schools in communities that permitted recreational cannabis dispensaries a year later in 2014, 3) high school students attending schools in 2015 in communities that have never allowed recreational cannabis dispensaries, and 4) high school students in 2015 attending schools in communities that permitted recreational cannabis dispensaries a year earlier in 2014 (Table 1).

**Table 1:**
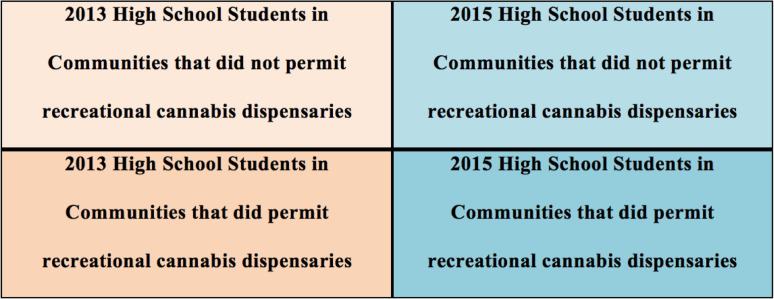
Four groups used in ANOVA study

## Results

There were statistically significant differences between groups in terms of “student use” as determined by one way ANOVA (F (3, 6038) = 29.931, *p* < .0001), statistically significant differences between groups in terms of “wrongfulness” as determined by one way ANOVA (F (3, 2926) = 16.865, *p* < .0001), statistically significant differences between groups in terms of “harmfulness” as determined by one way ANOVA (F (3, 2926) = 15.049, *p* < .0001), and statistically significant differences between groups in terms of “accessibility” as determined by one way ANOVA (F (3, 2923) = 32.158, *p* < .0001) (Tables 2, 3, 4, 5, 6, 7, 8, 9, 10, 11, 12 and 13).

**Table 2:** Cannabis Use Descriptives

Cannabis Use	N	Mean	Std. Dev.	Std. Error	95% Confidence Interval for Mean		Min	Max
					Lower Bound	Upper Bound		
No Permit 2013	1540	1.62	1.365	.035	1.55	1.68	1	6
No Permit 2015	1306	1.53	1.281	.035	1.46	1.60	1	6
Yes Permit 2013	1982	1.97	1.685	.038	1.90	2.05	1	6
Yes Permit 2015	1214	1.88	1.632	.047	1.79	1.97	1	6
Total	6042	1.77	1.526	.020	1.73	1.81	1	6

Cannabis Use in past 30 days

**Table 3:** Cannabis Use ANOVA

Cannabis Use	Sum of Squares	df	Mean Square	F	Sig.
Between Groups	206.218	3	68.739	29.931	.000
Within Groups	13,866.699	6038	2.297		
Total	14,072.917	6041

**Table 4:** Cannabis Use Post Hoc Tukey HSD

Dependent Variable Cannabis Use		Mean Difference I-J	Std. Error	Sig.	95% Confidence Interval	
I	J				Lower Bound	Upper Bound
No 2013	No 2015	.082	.057	.478	−.06	.23
	Yes 2013	−.358	.051	.000	−.49	−.23
	Yes 2015	−.265	.058	.000	−.41	−.12
No 2015	No 2013	−.082	.057	.478	−.23	.06
	Yes 2013	−.439	.054	.000	−.58	−.30
	Yes 2015	−.347	.060	.000	−.50	−.19
Yes 2013	No 2013	.358	.051	.000	.23	.49
	No 2015	.439	.054	.000	.30	.58
	Yes 2015	.092	.055	.338	−.05	.23
Yes 2015	No 2013	.265	.058	.000	.12	.41
	No 2015	.347	.060	.000	.19	.50
	Yes 2013	−.092	.055	.338	−.23	.05

**Table 5:** Ease of Access Descriptives

Access	N	Mean	Std. Dev.	Std. Error	95% Confidence Interval for Mean		Min	Max
					Lower Bound	Upper Bound		
No Permit 2013	731	2.76	1.225	.045	2.67	2.85	1	4
No Permit 2015	632	2.61	1.247	.050	2.51	2.71	1	4
Yes Permit 2013	991	2.31	1.153	.037	2.24	2.38	1	4
Yes Permit 2015	573	2.23	1.111	.046	2.13	2.32	1	4
Total	2927	2.47	1.203	.022	2.43	2.51	1	4

Perceived Ease of Access to Cannabis

**Table 6:** Ease of Access ANOVA

Access	Sum of Squares	df	Mean Square	F	Sig.
Between Groups	135.257	3	45.086	32.158	.000
Within Groups	4097.996	2923	1.402		
Total	4233.252	2926

**Table 7:** Ease of Access Post Hoc Tukey HSD

Dependent Variable Access		Mean Difference I-J	Std. Error	Sig.	95% Confidence Interval	
I	J				Lower Bound	Upper Bound
No 2013	No 2015	.154	.064	.078	−.01	.32
	Yes 2013	.455	.058	.000	.31	.60
	Yes 2015	.538	.066	.000	.37	.71
No 2015	No 2013	−.154	.064	.078	−.32	.01
	Yes 2013	.300	.060	.000	.15	.46
	Yes 2015	.384	.068	.000	.21	.56
Yes 2013	No 2013	−.455	.058	.000	−.60	−.31
	No 2015	−.300	.060	.000	−.46	−.15
	Yes 2015	.084	.062	.534	−.08	.24
Yes 2015	No 2013	−.538	.066	.000	−.71	−.37
	No 2015	−.384	.068	.000	−.56	−.21
	Yes 2013	−.084	.062	.534	−.24	.08

**Table 8:** Harmfulness Descriptives

Harmfulness	N	Mean	Std. Dev.	Std. Error	95% Confidence Interval for Mean		Min	Max
					Lower Bound	Upper Bound		
No Permit 2013	731	2.56	1.104	.041	2.48	2.64	1	4
No Permit 2015	635	2.54	1.073	.043	2.45	2.62	1	4
Yes Permit 2013	991	2.31	1.153	.037	2.24	2.38	1	4
Yes Permit 2015	573	2.23	1.111	.046	2.13	2.32	1	4
Total	2930	2.40	1.124	.021	2.36	2.45	1	4

Perceived Harmfulness of Cannabis

**Table 9:** Harmfulness ANOVA

Harmfulness	Sum of Squares	df	Mean Square	F	Sig.
Between Groups	56.187	3	18.729	15.049	.000
Within Groups	3641.556	2926	1.245		
Total	3697.742	2929

**Table 10:** Harmfulness Post Hoc Tukey HSD

Dependent Variable Harmfulness		Mean Difference I-J	Std. Error	Sig.	95% Confidence Interval	
I	J				Lower Bound	Upper Bound
No 2013	No 2015	.020	.061	.988	−.14	.18
	Yes 2013	.249	.054	.000	.11	.39
	Yes 2015	.333	.062	.000	.17	.49
No 2015	No 2013	−.020	.061	.988	−.18	.14
	Yes 2013	.230	.057	.000	.08	.38
	Yes 2015	.313	.064	.000	.15	.48
Yes 2013	No 2013	−.249	.054	.000	−.39	−.11
	No 2015	−.230	.057	.000	−.38	−.08
	Yes 2015	.084	.059	.481	−.07	.23
Yes 2015	No 2013	−.333	.062	.000	−.49	−.17
	No 2015	−.313	.064	.000	−.48	−.15
	Yes 2013	−.084	.059	.481	−.23	.07

**Table 11:** Wrongfulness Descriptives

Wrongfulness	N	Mean	Std. Dev.	Std. Error	95% Confidence Interval for Mean		Min	Max
					Lower Bound	Upper Bound		
No Permit 2013	731	2.29	1.089	.040	2.21	2.37	1	4
No Permit 2015	635	2.14	1.091	.043	2.05	2.22	1	4
Yes Permit 2013	991	2.52	1.155	.037	2.45	2.60	1	4
Yes Permit 2015	573	2.42	1.117	.047	2.32	2.51	1	4
Total	2930	2.36	1.127	.021	2.32	2.40	1	4

Perceived Wrongfulness of Cannabis

**Table 12:** Wrongfulness ANOVA

Wrongfulness	Sum of Squares	df	Mean Square	F	Sig.
Between Groups	63.195	3	21.065	16.865	.000
Within Groups	3654.769	2926	1.249		
Total	3717.965	2929

**Table 13:** Wrongfulness Post Hoc Tukey HSD

Dependent Variable Wrongfulness		Mean Difference I-J	Std. Error	Sig.	95% Confidence Interval	
I	J				Lower Bound	Upper Bound
No 2013	No 2015	.156	.061	.050	.00	.31
	Yes 2013	−.231	.054	.000	−.37	−.09
	Yes 2015	−.123	.062	.201	−.28	.04
No 2015	No 2013	−.156	.061	.050	−.31	.00
	Yes 2013	−.387	.057	.000	−.53	−.24
	Yes 2015	−.278	.064	.000	−.44	−.11
Yes 2013	No 2013	.231	.054	.000	.09	.37
	No 2015	.387	.057	.000	.24	.53
	Yes 2015	.108	.059	.251	−.04	.26
Yes 2015	No 2013	.123	.062	.201	−.04	.28
	No 2015	.278	.064	.000	.11	.44
	Yes 2013	−.108	.059	.251	−.26	.04

The Tukey HSD Post Hoc test calculated that high school students in communities that permitted recreational cannabis dispensaries in 2014 had a statistically significantly higher cannabis use than students in communities that did not permit recreational dispensaries. This occurred in both 2013 and 2015. Comparing between years, the results are within the margin of error and do not represent a statistically significant difference from 2013 to 2015.

Similarly, in terms of perceptions regarding how wrong cannabis use is, in both 2013 and 2015, high school students in communities that permitted recreational cannabis dispensaries had a statistically significant difference in their belief that cannabis use was less wrong than students in communities that did not permit recreational dispensaries. Furthermore, in communities that did not allow recreational dispensaries, a statistically significant difference was that students believed cannabis use was more wrong in 2015 than in 2013. In communities that permitted recreational dispensaries, the results are within the margin of error and do not represent a statistically significant difference from 2015 to 2013.

Regarding how harmful students perceived the regular use of cannabis to be, there was a statistically significant difference between the two types of communities in both 2013 and 2015. Students in communities that permitted recreational dispensaries believed that regular cannabis use was less harmful than students in communities that did not allow recreational dispensaries. Comparing between years, the results are within the margin of error and do not represent a statistically significant difference from 2013 to 2015.

In terms of ease of access in obtaining cannabis, there was a statistically significant difference between students in the two types of communities in both 2013 and 2015. Students in communities that permitted recreational dispensaries believed that cannabis was more difficult to obtain than students in communities that did not allow recreational dispensaries. Comparing between years, the results are within the margin of error and do not represent a statistically significant difference from 2013 to 2015.

## Discussion

In both 2013 and 2015, students in communities that permitted recreational dispensaries used more cannabis, thought cannabis was less harmful, less wrong, and was more difficult to access than high school students in communities that did not permit recreational cannabis dispensaries. A possible explanation for this difference is that the high school students mirrored the behavior and perceptions of the adult population of their communities. By vote or by representation, the adults in these communities had decided to approve or ban recreational cannabis dispensaries; a reasonable conclusion from this could be that the adults in a community that permitted recreational cannabis dispensaries would use more cannabis, believe it was less harmful and wrong, and might perceive cannabis more difficult to access than those adults in communities that chose not to permit recreational dispensaries.

Another statistically significant finding was that in communities that did not permit recreational dispensaries, the students thought cannabis use was more wrong in 2015 than in 2013. More studies are needed to determine the cause of this change. The difference between 2013 and 2015, in terms of use, harm, accessibility, and the difference in wrongfulness in communities permitting recreational dispensaries did not achieve a statistically significant difference, however, across all types of communities the trend from 2013 to 2015 was that high school cannabis use declined, was thought of as more wrong, more harmful, and was more accessible. With the 2017 test being administered in fall 2017, it will be interesting to see if the trend continues and, if the results will be statistically significant. Why there is a shift in behavior and attitude, or why there is no shift will need to be studied.

## Limitations

There were multiple limitations to our study. The study sample was self-selecting and students in private schools, alternative schools, or youth not attending school were not included. Also, even though students were assured their responses were confidential and anonymous, the data collected was self-reported and respondents may have inaccurately reported their cannabis use or perceptions towards cannabis. This study focused on only southcentral Colorado and was limited to only 7 communities and 12 high schools. The results may not represent the full region or the state. Furthermore, as this study used data collected from a cross-sectional survey, the HKCS, the data provides a snapshot in time and not a longitudinal study of a group over time. The results cannot be used to determine causal relationships but they may be used to make inferences about possible relationships.

## Conclusions

Based on the 2013 and 2015 Healthy Kids Colorado Survey data, permitting recreational cannabis dispensaries in a community does not appear to change student cannabis use or perceptions towards cannabis. Future studies are recommended to corroborate these results. One proposed study is a survey of school administrators that collects data on cannabis violations in middle and high schools to see if the school reported data aligns with the student self-reported data. Another study could determine the level of cannabis prevention education in a district to determine if education has an effect on usage and perceptions.

## Abbreviations

ANOVA: Analysis of variance
HKCS: Healthy Kids Colorado Survey
HSD: Honestly Significant Difference

## References

[CR1] Brooks-Russell A, Johnson R, Ma M, Kattari L, Anderson Goodell EM, Kirchner T, Levinson A (2018). Adolescent marijuana use before and after legalized recreational marijuana in Colorado.

[CR2] Brooks-Russell A, Ma M, Levinson AH, Kattari L, Kirchner T, Anderson Goodell EM, Johnson RM. Adolescent marijuana use, marijuana-related perceptions, and use of other substances before and after initiation of retail marijuana sales in Colorado (2013-2015). Prev Sci. 2017, 2018; PMID: 30043198. 10.1007/s11121-018-0933-2 PMC8086773 30043198

[CR3] Colorado Department of Public Health and Environment (2016). Healthy kids Colorado survey and smart source information.

[CR4] Harpin SB, Brooks-Russell A, Ma M, James K, Levinson A (2018). Adolescent marijuana use and perceived ease of access before and after recreational marijuana implementation in Colorado. Subst Use & Misuse.

[CR5] Hasin DS, Wall M, Keyes K, Cerda M, Schulenberg J, O’Malley PM, Galea S, Pacula R, Feng T (2015). Medical marijuana laws and adolescent marijuana use in the USA from 1991 to 2014: results from annual, repeated cross-sectional surveys. Lancet Psychiatry.

[CR6] Hinckley S (2016) Pot is legal in Massachusetts. What do opposing towns do now? The Christian Science Monitor. 18 Nov. 2016. http://www.csmonitor.com/USA/Society/2016/1118/Pot-is-legal-in-Massachusetts.-What-do-opposing-towns-do-now. Accessed 14 June 2017.

[CR7] Johnson J, Hodgkin D, Harris SK (2017). The design of medical marijuana laws and adolescent use and heavy use of marijuana: Analysis of 45 states from 1991 to 2011. Drug Alcohol Depend.

[CR8] Light M, Orens A, Rowberry J, Saloga C (2016) The economic impact of marijuana legalization in Colorado. Marijuana Policy Group, Marijuana Policy Group, Oct 2016 http://www.mjpolicygroup.com/pubs/MPG%20Impact%20of%20Marijuana%20on%20Colorado-Final.pdf. Accessed 25 June 2017. Colorado. Marijuana Policy Group. Retrieved 11 May 2017.

[CR9] Mooney AM (2016) Fifteen Colorado Counties Will Vote on Marijuana Measures in November. Westword, 9 Nov. 2016. http://www.westword.com/marijuana/fifteen-colorado-counties-will-vote-on-marijuana-measures-in-november-8394122. Accessed 30 June 2017.

[CR10] Morris RG, TenEyck M, Barnes JC, Kovandzic TV (2014). The effect of medical marijuana Laws on crime: evidence from state panel data, 1990-2006. PLoS One.

[CR11] State of Colorado (2017). Marijuana Tax Data. Department of Revenue.

[CR12] Wallace A (2016) Pueblo voters reject proposals to shut down existing marijuana businesses. The Cannabist. 9 Nov. 2016. http://www.thecannabist.co/2016/11/09/pueblo-county-colorado-question-200-marijuana-question-300/66898/. Accessed 30 Aug. 2017.

[CR13] Westrope A (2017) Roseville resumes recreational marijuana debate. The Press Tribune Newspaper. 19 May 2017. https://goldcountrymedia.com/live-content/the-press-tribune/. Accessed 16 June 2017.

[CR14] Wilder Z (2017) “14 more states that might legalize Cannabis this year - culture.” MERRY JANE. 18 Feb. 2017. http://merryjane.com/culture/states-that-might-legalize-weed-2017. Accessed 21 June 2017.

[CR15] Wilson J (2015). The libertarian argument for legalizing marijuana. A libertarian future, 6 June 2015.

